# Irregularity

**Published:** 1873-05

**Authors:** Isaiah Forbes


### SELECTED ARTICLES.

### ARTICLE IV.

### *Irregularity.*

BY ISAIAH FORBES, D. D. S.

Writing upon the subject of the causes that produce irregularity, if one coincides with the generally expressed opinions of the day, would be a waste of time, and the result an unprofitable paper, for the subject seems to be completely exhausted. But when the subject is to be considered in a different light from that in which our early education has taught us, a new field is open for our consideration, reflection and investigation. I will therefore confine myself to that branch of the subject which we are educated to regard as the principal cause of irregularity of the dental arch, viz.: the premature extraction of the deciduous teeth—and while expressing my well known convictions on this subject, I cannot but feel that my position is an unenviable one. There is nothing so difficult to change as the impressions and prejudices of our early education. The more I have studied and reflected on this branch of the subject, the more have I become satisfied that the doctrine, as taught in our dental text books, cannot be correct. Several years have passed since I first expressed my convictions on this subject in our local societies, and the most *forcible* argument that was then advanced in opposition was: "Is that the way you do in your practice?"

Some two years after this period, while opposing a paper on the subject of irregularity, I had the proud satisfaction of learning that Mr. Tomes in his histological investigations had come to the same conclusion. In his "System of Dental Surgery" he says—"It has been usual to assume that the prematnre extraction of the temporary teeth occasions a contraction of the jaw, but I do not think that any anatomical facts can be brought forward in support of the supposition. If a temporary tooth be removed, the crowns of the contiguous teeth may lean toward each other and give the appearance of contraction, but it does not really involve a diminished size of that part of the jaw from which the tooth has been lost."

Again: (page 188.) "If specimens be examined in which the two sets of teeth are present, it will be seen that the implantation of the temporary teeth occupies but a very small space in the alveolar ridge, as compared with that occupied by the crowns of the permanent teeth. Organs in an active state of development induce the expansion of the parts about them, and there is no good reason for supposing that the jaw forms an exception to this rule. The persistence of the first, which are placed immediately in front of the second, still may, and frequently does interfere with the outer progress of the latter; but I cannot see how the removal of the temporary can produce a prejudicial influence upon the arrangement of the permanent teeth." "In the case from which the accompanying illustration is taken, the two central incisors were lost long before their successors were ready for eruption, hence the sockets became obliterated, and the alveolar ridge made good; but we do not see the slightest trace of contraction of the jaw." This case presents so strong a similarity to one in my own family that I present it as another proof of the position I have taken. No matter how much deference a dentist may pay to the prejudices of early education in his general practice, he is at liberty to exercise his own judgment or convictions in his own family, without the fear of compromising the dignity of his profession among his brethren. My elder children *played* dentistry on my youngest by using Excavators, and kept at it until, subsequently, the incisors became painful, before I was aware of what had taken place; and the young rascal was so stubborn that he would not even permit his mother to make an application for his relief, when she concluded, for the comfort of the child, that the teeth had better be extracted. As soon as I was made acquainted with the facts, out the teeth came before I let go of his head, both centrals and one lateral; now, *his jaw* and the permanent teeth are as well developed as if the temporary teeth had been allowed to remain until the roots had become completely absorbed. Prof. Gross tersely says, "There is generally a great prejudice, even on the part of dentists, against the extraction of the deciduous teeth, on the supposition that it has a tendency to interfere with the development of the permanent set. I have been at much pains to inquire into the matter, and am satisfied that the idea is altogether erroneous. On the contrary, the operation, so far from being injurious, will generally be found to be eminently beneficial, not only relieving pain, but conducing to the beauty and perfection of the future organs." Thus briefly does he dismiss the subject, clearly intimating that he who becomes thoroughly acquainted with anatomy, the development of the jaws and the teeth, must be convinced that the theory held by many, and taught in our text books, must be incorrect.

One of the great causes of irregularity is the retention of the temporary teeth beyond the proper period for their shedding; the lateral pressure of the permanent incisors and the bicuspid against the temporary canine has retained that tooth beyond its proper period, and diminished the prospect for the development of the canine, and in some cases entirely obliterated the membranes that furnish the ossific material for the development of the tooth. The jaw grows and expands by interstitial lamellæ, the same as the softer tissues. Common sense *indicates* it. Anatomy and physiology proclaim it. Histology and microscopy confirm it. Let us be ready to investigate the errors of our previous education and acknowledge the truth as made manifest by the researches of the ardent student. We are living in too enlightened an age to declare or sustain the characters of the persecutors of a Harvey.

The more a student studies osteology, and the better he becomes familiarized with the structure and growth of bones, the more will he become convinced that the premature extraction of the deciduous teeth cannot possibly cause any contraction of, or diminish *in any sense the growth* of the *jaw.* Different arteries, or branches of the same artery supply the teeth, the alveolus, and the jaw. While doing so neither is dependent on the other for nourishment. The alveolus is formed during the growth of the roots of the teeth, yet not dependent on the blood vessels of the teeth for nourishment, and when the deciduous teeth are extracted, the blood vessels supplying the jaw are not in any manner contracted, nor the ossific material they may have contained diminished. The facial artery supplies the jaw, the alveolus and the teeth ; the alveola grows with the growth of the tooth, the growth of the alveolus becoming necessary for the support of the tooth. Extract the tooth, its *support* not being wanted in the animal economy is dissolved away, neither of which, when lost to the mouth, can effect a contraction of the arteries that ramify through the jaw and supply it with ossific material. When a tooth is extracted it is simply, as it were, clipping off the end of an artery that supplied the tooth, and not the artery that supplies the jaw, and if any change *is effected*, science must decide that the *retention* of the temporary tooth would have the tendency to contract the jaw, for the blood that flows to nourish the teeth and process would then be free to nourish and develop the jaw ; nor can I see how mastication of the food by the temporary teeth can *increase* the flow of the blood to the jaw, or the want of the *means* of *mastication diminish* the flow of the blood to the jaw, except in so far as want of proper mastication of the food may lengthen the time for chymification, and consequently, less chyle becomes assimilated with the blood for the time being, and then one portion of the system suffers no more than another. Again, it is claimed that the food should be well masticated to obtain a plentiful supply of saliva to mingle with the food, *to assist as a solvent.* Its importance in that particular is very questionable. The saliva is required simply for a lubricator, water answering the same purpose. It is certain that the saliva dissolves starch and sugar, but even these articles, when taken in the mouth, are not usually dissolved before deglutition takes place, and the more active dissolvent properties of the gastric juice does not require its assistance; in fact it has been proven by experiment, "that the mixture of the saliva with the gastric juice rather retarded its solvent action. I do not wish to deny the utility of the saliva; it is certainly important as a preliminary to digestion; its legitimate and only use is to lubricate the food, and to facilitate the passage of the bolus through the organs of deglutition. Water will answer the purpose nearly as well as saliva, though the mucous properties of this secretion may give it a slight preference."

Mr. Garretson, in his work on "Oral Surgery," speaking of crowded dentures, from the want of space in the arch, says: "This anomaly has, perhaps, the largest associative pathological condition. I remarked that this lesion, if we may term it such, is more frequently the fault of the surgeon than of nature. If, for one moment, we refer to certain physiological relations existing between the first and second dentures, we may find that it is within our power to prevent the many ills that follow so frequently in his train, and simply by doing little, or, more commonly, nothing. The deciduous arch, as we are all aware, is filled completely by its ten teeth. The second or permanent set is to comprise in number sixteen, and each tooth certainly quite as large as its predecessor. This increase in number and size of the teeth, it is evident, must be provided for in an enlargement of the alveolar arch. This provision is always attempted by nature in the process described by the physiologist as the elongatory. I will illustrate this process of maxillary enlargement by considering the ten milk teeth as so many wedges placed in a *springy* arch. This arch it is designed to lengthen by additions to either end. If, now, these wedges should be removed before others were ready to take their places, it is evident that the elongation being made at the ends, would, to a greater or less extent, be counterbalanced by the springing together of the parts at the sites of the removed wedges. The process of maxillary, or rather alveolar absorption is truly represented by this retraction of an arch. In proportion to the number of the deciduous teeth removed prematurely will be the curtailment in size of that arch, at least of its alveolar face.

"Prof. Gross and Mr. Tomes, also, in his last book, denied this position; why, I know not. With the greatest possible respect for the opinion of these gentlemen, it is my experience that they are wrong.

"If there is a pathological Pandora's box, it is certainly the lesion of an overcrowded maxillary arch. Such condition is made evident to the practitioner the moment he looks into the mouth of the patient; the teeth are jammed into the most uncomfortable looking positions; the deformity, however, mostly existing in the front of the mouth—either the central incisors override, or the laterals are thrown back, or otherwise the cuspidati take the tusk position, standing out prominently from the arch, the bicuspids occupying too anterior a location, approximating, indeed, not unfrequently with the lateral incisors."

Mr. Garretson says: "If we refer to certain physiological relations existing between the first and second dentures, we may prevent the many ills that follow so frequently in this train, and simply by doing little, or, more commonly nothing;" and yet he does not inform us what those physiological relations are, but simply gives us what he calls an illustration of the process of maxillary enlargement, which is described by "Physiologists as an elongatory; he considers the ten milk teeth as "so many wedges in a *springy* arch. This arch it is designed to lengthen by additions at either end," meaning to lengthen from the second deciduous molar to the ramus of the jaw. "If, now, these *wedges* should be removed before others were ready to take their place"—is it evident that the elongation being made at the ends would, to a greater or less extent, be counterbalanced by the springing together of the parts at the sites of the removed wedges, supposing one or more of the deciduous teeth had been prematurely extracted. After studying Wilson and Grey I cannot conceive how the premature extraction of the deciduous incisors could prevent the elongatory development of the jaw from the second deciduous molar to the ramus. Nor can I conceive how the *elongatory* enlargement of the jaw can make room for the *six front permanent teeth*, when they are "nearly as broad again" as the deciduous teeth, and if this "process of maxillary, or rather, alveolar absorption is *truly* represented by this retraction of an arch," I cannot conceive how 'tis possible to avoid irregularity of the front teeth.

We know that the first permanent or sixth year molars stand farther apart at the age of twenty than they did when first erupted, and they stand as perpendicular in the jaw. We know that the labial surface of the crowns of the six front teeth stand almost as perpendicularly in the adult as the deciduous teeth in the child; therefore the "elongatory process of enlargement" above, and the retention of the "deciduous wedges in the springy arch," would not, could not possibly increase the span of the arch "half as much again," so as to enlarge the space necessary to permit the teeth to attain a perfectly regular position. We know the six front *permanent* teeth are certainly quite as large again as their deciduous predecessors. We *know* the bicuspids are no larger than the deciduous molars. We *know* that in the incipient stages of development the permanent teeth lay, as it were, folded upon each other or lapping one another. We *know* that if the arch of the jaw did not enlarge, that the permanent teeth must erupt, and continue in an irregular position. We *know* that the base of the arch of a child's jaw is not as broad as a man's. We *know* that the child's tongue holds the same relation, as regards size, to the child's jaw as the man's tongue does to the man's jaw. We know that the man's tongue is much broader than the child's, therefore, the base of the arch of the man's jaw must be broader than the child's. We *know* that the *arch* of an adult jaw extends to the distal surface of the canines, from thence backward the jaw is nearly a straight line. We *know* that arterial branches, totally distinct and independent of each other, supply the jaws, the alveolus and the teeth. We *know* that the extraction of a temporary tooth, or the absorption of the temporary alveolus does not diminish the size of the Haversian canals through which the blood vessels flow that supply the permanent teeth and alveolus. We *know* that the Haversian canals, which are channeled out of this compact substance, convey the blood vessels for its nutrition. Therefore I cannot conceive how it is possible that the removal of one or more of "the wedges that compose the springy arch" would cause a "springing together of the parts at the sites of the removed wedges." On the contrary I can conceive how an *undue retention* of the deciduous teeth, and a firm alveolus on the labial surface of the jaw, might cause a "pathological pandora's box."

If my statements are anatomical and physiological truths, and cannot be controverted, let us be ready and willing to acknowledge, *a la* Harvey, that the blood *does* flow through the system, and reverse our advice to mothers when their little children are presented to us for relief, with badly decayed, aching teeth. Extract the teeth, thereby giving the child permanent relief, and say to the mothers—what appears to me to be the honest truth—"the extraction of the teeth cannot possibly do any harm, but on the contrary, even though by an application we could alleviate the pain, their retension might injure the healthy growth of the permanent teeth."—*Missouri Dental Journal.*

